# Cold atmospheric plasma effectively kills chordoma cells through induction of intracellular reactive oxygen species

**DOI:** 10.1038/s41598-025-05916-y

**Published:** 2025-07-01

**Authors:** Sophie Peeters, Peter B. Wu, Blake Haist, Amber Armellini, Wi Jin Kim, Zhitong Chen, Richard Obenchain, George Ayad, Weihong Ge, Aparna Bhaduri, Graeme Sabiston, Robert M. Prins, Richard Wirz, Anthony C. Wang

**Affiliations:** 1 Department of Neurosurgery, David Geffen School of Medicine, University of California Los Angeles, Los Angeles, CA USA; 2 Department of Mechanical and Aerospace Engineering, University of California Los Angeles, Los Angeles, CA USA; 3 Department of Mechanical, Industrial, and Manufacturing Engineering, Oregon State University, Corvallis, OR USA; 4 Department of Biological Chemistry, David Geffen School of Medicine, University of California Los Angeles, Los Angeles, CA USA; 5 College of Engineering, Oregon State University, Corvallis, OR USA

**Keywords:** Chordoma, Cold atmospheric plasma, Reactive oxygen species, CNS cancer, Astrophysical plasmas

## Abstract

Chordomas remain one of the most difficult-to-treat of skull base tumors. The best chance of survival and cure to date is with *en bloc* radical surgical resection, followed by adjuvant radiotherapy. Oftentimes, tumor infiltration into functionally critical, or difficult-to-access areas, precludes optimal resection. The median overall survival for these tumors is 116 months. Cold atmospheric plasma (CAP) is generated by applying a high voltage electric field to helium or argon feed gases, resulting in reactive atmospheric species. Over the past decade, CAP has been applied experimentally in a number of oncologic conditions and has demonstrated anti-tumor effects both in vitro and in vivo. Additionally, CAP has been shown to increase cancer cells’ sensitivity to radiation and could therefore be a useful tool in improving chordoma recurrence rates as an intraoperative adjuvant therapy to the current standard of care. To date, there are no studies in the literature examining the efficacy of CAP in inducing cytotoxicity in chordoma. We treated CH2, CH7 and UM-Chor1 chordoma cells with CAP, measuring resulting cell viability and intracellular ROS accumulation. Here, we show a dose-dependent increase in intracellular ROS and cell death with direct CAP exposure in vitro, finding an exquisite sensitivity of chordoma cells to CAP-mediated cytotoxicity.

**Supplementary Information:**

The online version contains supplementary material available at 10.1038/s41598-025-05916-y.

## Introduction

Chordomas are slow-growing malignant tumors most commonly arising in the clivus or sacrum^1^. Derived from notochord remnants, chordomas tend to be locally aggressive, particularly in the clivus, and often impact critical nearby structures such as the internal carotid arteries and cranial nerves mediating eye movements and facial sensation^2^. Due to their low prevalence, there is limited clinical data regarding skull base chordomas and their pathophysiology and treatment. In the United States of America, the incidence of chordoma is 0.06 to 0.1 per 100,000 people^3^. Average age at presentation for skull base chordomas is 47.4 years, and average age at presentation for sacral chordomas is 62.7 years. Management of skull base chordoma requires multi-modal care, with the current gold standard treatment for these lesions including surgical resection, most often followed by adjuvant radiation irrespective of recurrence^4,5^. However, the most important predictor of outcome (local tumor control, overall and progression free survival) remains the extent of resection, ideally *en bloc* with wide or marginal negative margins^6^. In the skull base, *en bloc* resection is typically impossible, given the infiltrative nature of the disease, the small access corridor for surgery, and the critical nature of nearby structures in the skull base. With residual chordoma, the recurrence rate is dramatically elevated, despite adjuvant radiotherapy administration.

The efficacy and role of adjuvant radiotherapy options is debated. A number of studies have not demonstrated significant improvement in survival with the addition of adjuvant radiation following surgery^2,5,7^. The preferred option is currently high-dose heavy particle therapy (such as proton beam or carbon ion radiation) due to a favorable Bragg peak effect allowing for sharp margins^2^. A meta-analysis concluded a more favorable progression free survival with carbon ion radiotherapy compared to stereotactic radiosurgery, but no significant overall survival difference^6^. According to a 2022 meta-analysis, the 5-year local control rate, overall survival, and progression-free survival for skull base chordomas treated with proton beam therapy as primary or adjuvant therapy were 76.6%, 79.6% and 89% respectively^5^. Meanwhile, the photon therapy 5-year local control rates were reported as 33.5% with overall survival at 53.5%^8^. Radiosurgery 5-year local control rate is reported to be 56%, however, overall survival at 75% was comparable to proton beam therapy^5^. Despite its focused nature, photon radiotherapy still causes destruction of healthy tissue beyond the targeted volume^9^. The most severe side effects include hearing loss, parenchymal necrosis, visual disturbances, and pituitary insufficiency^5^. This risk is slightly mitigated with charged particle or hadron therapy (i.e., carbon ion beam therapy) as an alternative, which deposits energy at a higher rate and more precisely than photons, and compared to photon radiotherapy, is associated with improved control rates^9–11^. The relatively high recurrence rates in chordomas, despite maximal surgical resection and adjuvant radiotherapy, demands alternative adjuvant options^12^.

Cold atmospheric plasma (CAP) is generated through high voltage electrical field stimulation of helium or argon feed gases, resulting in reactive atmospheric species^13–15^. CAP has been shown to selectively kill rapidly-dividing cells through both chemical factors (reactive species-mediated oxidative stress activating the apoptosis cascade), as well as physical factors (electromagnetic forces leading to cell membrane disruption and eventual necrosis)^16^. The reactive oxygen and nitrogen species (RONS) generated by CAP exposure lead to inflammatory changes and cytotoxicity in cells exposed to adequately high doses^17–19^. In addition to RONS-mediated apoptotic cell death, CAP has been shown to lead to immunogenic cell death, as well as increase sensitivity to other external stressors, such as cancer therapies^20^.

CAP has emerged as a promising new modality of cancer therapy, triggering selective tumor cell death in lung, breast, ovarian, head and neck, and gastrointestinal cancers, amongst others, with minimal impacts upon surrounding healthy tissues^17,20–23^. More recently, some groups, including our own, have shown successful CAP-mediated cell death in glioma cells as well^21,24^. In vivo xenograft studies have also suggested increased survival and decreased tumor size after CAP treatment in bladder, skin, breast, lung and colon preclinical cancer models^16^. Three clinical trials have employed CAP as an anti-cancer therapy: one in metastatic solid tumor patients, one for palliative use in advanced squamous cell head and neck carcinomas, and one for cervical intraepithelial neoplasia^25–28^. There have been no studies to date in the literature investigating the effect of CAP on chordoma.

There is a dire need for effective therapies targeting residual unresected chordoma in a tissue-selective manner. The ability to directly deliver CAP to microscopic residual intraoperatively with inherent tumor selectivity makes CAP an attractive novel chordoma therapy. In this study, we demonstrate that (1) CAP leads to cytotoxic levels of intracellular accumulation of reactive oxygen species (ROS) in chordoma cells in vitro, (2) ROS accumulation contributes to CAP-mediated cell death, (3) duration of plasma treatment is directly linked to extent of cell death and intracellular ROS accumulation in a dose-dependent manner.

## Methods

### Cold atmospheric plasma generation

The CAP used for our experiments is delivered via a custom atmospheric plasma jet^29,30^. As shown in supplementary Fig. 1, the plasma jet uses a single-electrode design to provide highly localized delivery of CAP species to the target^31^. The jet is fed with high purity helium gas at a constant flow rate of 1.21 L/min. The sharpened copper electrode is supplied with 1.4 kV peak-to-peak sinusoidal voltage at 52 kHz. The high voltage, high frequency electrical field is supplied by a BK Precision 4013 DDS Sweep Function Generator and CPX400DP-Dual 420 W PowerFlex Direct Current power supply unit with custom step-up electronics. The primary plasma discharge takes place at the electrode tip, and the CAP species flow downstream to the target, as shown diagrammatically in Supplementary Fig. 1 (inset image shows the free-space evolution of the jet during operation). The end of the pipette tip was consistently maintained one centimeter above the samples during delivery in all experiments. The target cells were treated with CAP for 0, 10, 30, 60, 90 and 180 s, with at biological triplicate for each condition.

### Cell culture

We used 3 different human chordoma cell lines for our experiments: UM-Chor1 (ATCC CRL-3270), CH2 (ATCC CRL-3218), and CH7 (ATCC CRL-3404). The cells were cultured in 4:1 Isocove Modified Dulbecco’s Medium (ATCC 30–2005) and RPMI-1640 Medium (ATCC 30–2001) supplemented with 10% fetal bovine serum (ATCC). Cell cultures were maintained in a humidified incubator at 37 °C with 5% C0_2_ and analyzed daily under brightfield microscopy.

### Cell viability assays

Cell viability was measured using the Cell Titer Glo assay (Promega, G7570), which is a luminescent assay to determine the number of viable cells in culture. The assay quantitates adenosine triphosphate (ATP) as a marker of metabolically active cells. Once the reagent is added, cell lysis occurs and a luminescent signal proportional to the amount of ATP present is generated.

Cells were plated into opaque white 96-well plates at a density of about 20,000 cells per well in 100 uL of media. Confluence was ensured to be about 20%. In order to ensure adherence and stability, the cells were incubated for one day after plating. The media was then replaced the following day and the cells were treated with CAP. Subsequently, the cells were again incubated at 37 °C for 48 h. The Cell Titer Glo reagent mixture was then added to all the cells (100uL per well). After 2 min of orbital shaking of the plate and 10 min of incubation for equilibration, the luminescence signal was then recorded using a SpectraMax microplate reader. The entire set of experiments was repeated three times in triplicate.

### Intracellular ROS measurement

We used the cell-based quantitative Cellular ROS Assay (Abcam; ab186027). The assay uses a cell-permeable red dye that generates red fluorescence upon reacting with ROS in live cells. Cells were plated into black clear-bottom 96-well plates at a density of about 20,000 cells/well in 100uL of media. Confluence was ensured to be about 20%. In order to ensure adherence and stability, the cells were incubated for one day after plating. The media was then replaced the following day, and 100uL of the ROS Red Stain working solution (see detailed Abcam protocol for working solution) was added to each well. The cells were then incubated 30–60 min at 37 °C and subsequently treated with CAP at various durations. The cells were then incubated again, and fluorescence was measured at 12, 24 and 48 h using a SpectraMax microplate reader (Ex/Em = 520/605 nm). The ROS change is represented as a percentage of the control value after subtraction of the background signal.

### Statistical analysis

Student’s t-test was performed to test for significance (*p <.05, **p <.01, ***p <.005, ****p <.001). Results were plotted using a Microsoft Excel Software with mean +/- standard deviation and standard error of the mean.

## Results

### Intracellular ROS accumulation

**Fig. 1:**
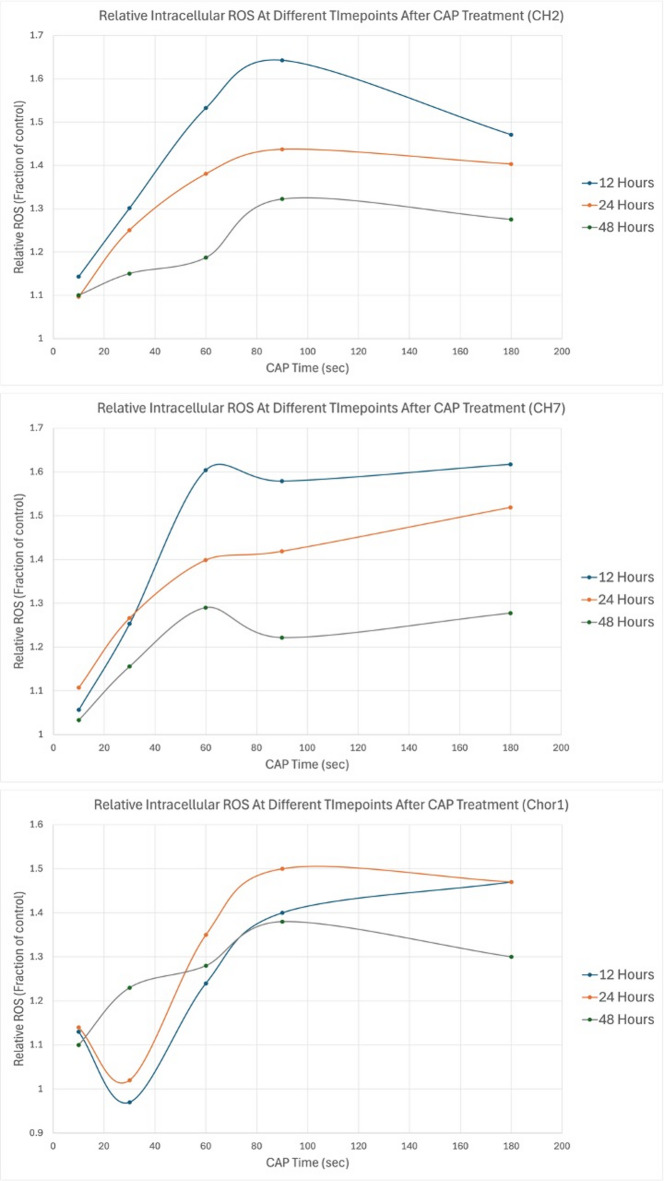
Intracellular ROS relative to untreated control cells at 3 different timepoints (12, 24 and 48 h) for three different chordoma cell lines after various durations of CAP treatment (top to bottom: CH2, CH7, and Chor1).

Intracellular accumulation of ROS was noted in chordoma cells after defined durations of CAP treatment. The relative concentration of ROS compared to untreated control cells increased with longer CAP treatment times at all three time points (12, 24, and 48 h) for all studied cell lines (Fig. 1).

### Cell survival based on plasma treatment duration

**Fig. 2:**
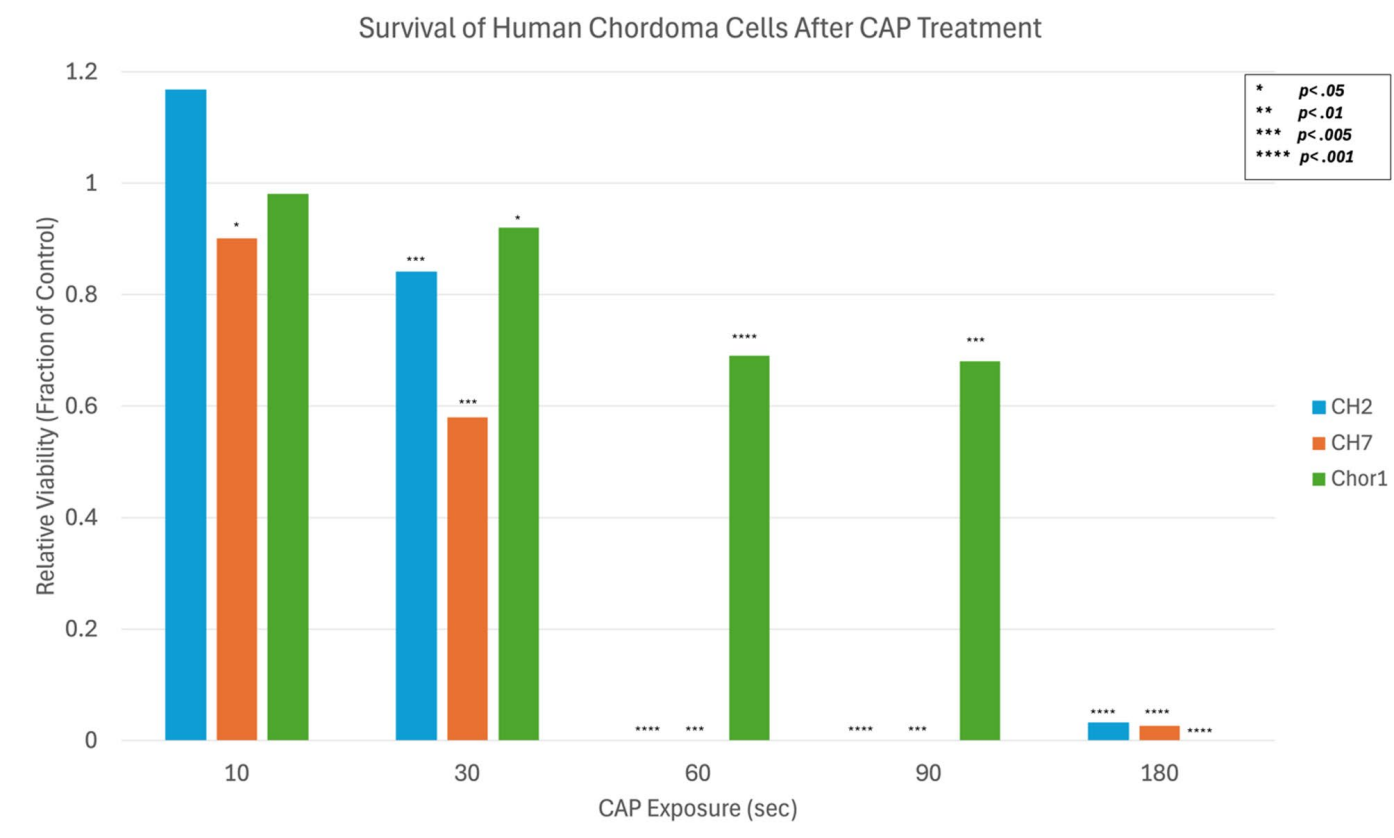
Percent viability of human chordoma cells after various lengths of direct CAP treatment. Significance measured compared to untreated control (*p <.05, **p <.01, ***p <.005, ****p <.001).

After various durations of direct CAP treatment, chordoma cells consistently demonstrate a dose-dependent decrease in viability with longer CAP exposure leading to greater cell death (Fig. 2). This effect was noted in all 3 chordoma cell lines. The degree of cytotoxicity was significant compared to untreated control cells at 30 s or more for all three cell lines (with p values < 0.05 in all cases). The IC50 was between 30 and 60 s for the CH2 and CH7 cells, and approximately 100 s for UM-Chor-1 cells.

### Intracellular ROS concentration

**Fig. 3:**
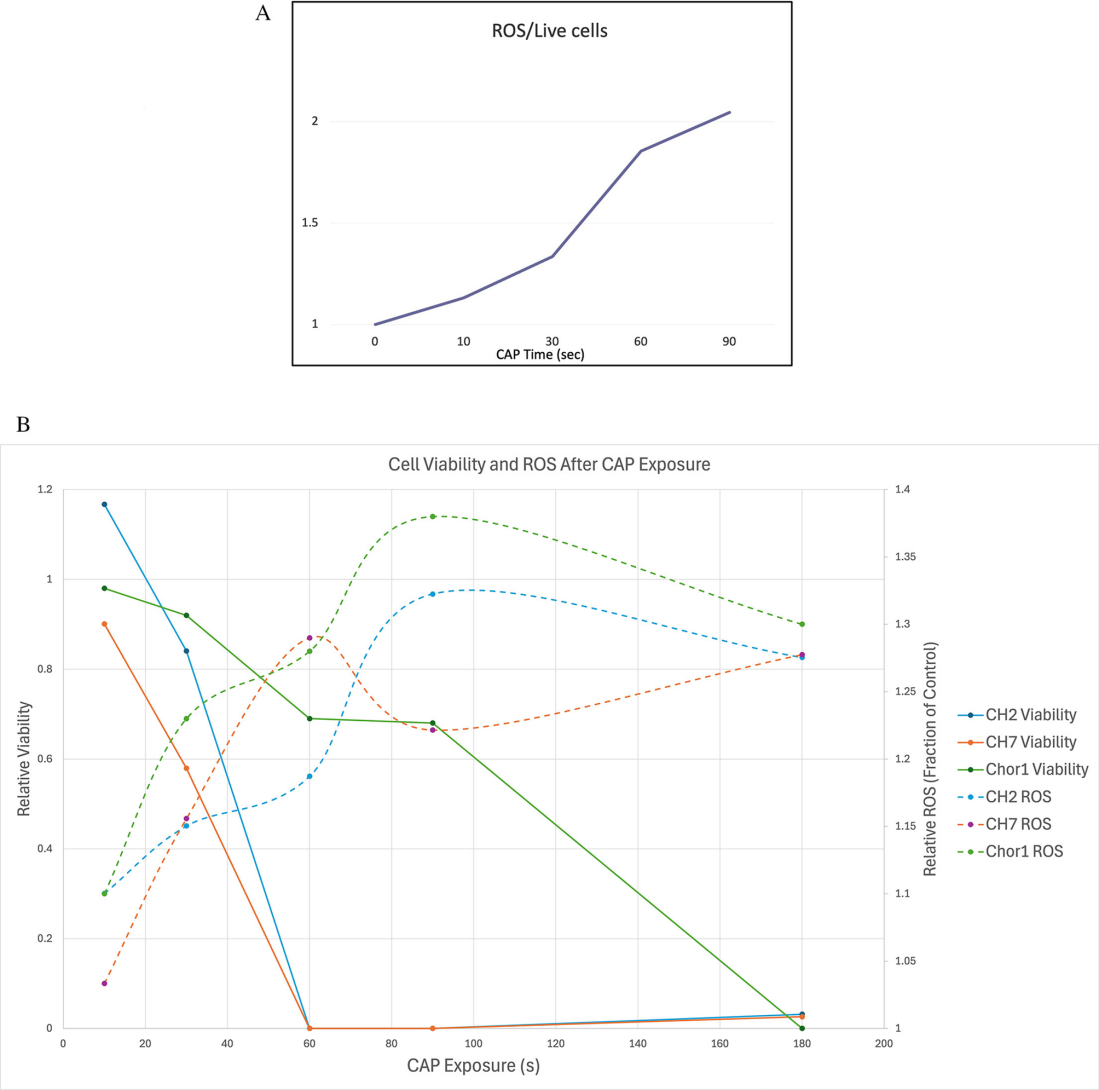
(**A**) Ratio of intracellular ROS to live cells compared to untreated control in UM-Chor-1 chordoma cells. (**B**) Cell viability and correlating ROS accumulation based on different lengths of CAP exposure for all 3 chordoma cell lines (CH2, CH7, and UM-Chor-1).

With cell viability significantly decreasing with longer CAP exposure, we plotted the ratio of intracellular ROS accumulation to live cells in the UM-Chor-1 cell line, which doubles by 90 s of CAP treatment compared to untreated cells (Fig. 3A). This ratio could not be calculated for the other two cell lines given the number of surviving cells was zero for both by 60 s of CAP treatment. To try and provide as similar an analysis as possible, we illustrate the opposing survival and ROS trends with increased CAP treatment times (Fig. 3B). Thus, with increased direct CAP exposure, there is an increase in ROS intracellular concentration.

## Discussion

Chordomas remain very challenging to treat, with high recurrence rates despite current standard of care multi-modal therapy. Achieving *en bloc* or radical surgical resection is often not feasible in skull base chordomas, and adjuvant radiotherapy is too frequently ineffective in preventing local recurrence. There are few promising adjuvant therapies on the horizon for these types of locally invasive tumors. CAP has been effective in selectively killing various cancer types in vitro and in vivo, and is being tested in ongoing clinical trials for head and neck cancers and metastatic solid tumors^16^.

Here, we observed dose-dependent cell death in vitro in three different human chordoma cell lines with increasing doses of direct CAP exposure. The average tumor cell death rate was superior to reported in vitro studies of CAP-treated glioma cells, with an IC50 being between 90 and 120 s versus 30–60 s for two of our three chordoma cell lines, and 100 s for the third^32^. Chordoma being a slow-growing malignancy of mesodermal origin, this finding suggests that proliferation rate is not the only factor in determining responsiveness to CAP treatment, as suggested by Keidar, et al.^22^. With regards to the mechanism of CAP-induced cell death in chordoma cells, we demonstrated a dose-dependent increase in intracellular ROS accumulation with increasing CAP treatment time, with a commensurate decrease in chordoma cell viability. Presumably, chordoma cells are sensitive to ROS-mediated cell death, a possibility that has not been widely studied. Further investigation is needed to elucidate the factors underpinning the sensitivity of chordoma cells to RONS-mediated cell death mechanisms.

Generally, the best understood mechanism of CAP-mediated cell death in cancer is RONS-mediated cytotoxicity, which has been demonstrated both in vitro and in vivo, inducing cell cycle arrest and conferring survival benefits in xenograft mouse models^33–35^. Moreover, multiple papers have identified signs of immunogenic cell death in CAP-treated tumor cells, both in vitro and in vivo, which is thought to occur through the emission of damage-associated molecular pattern (DAMP) signaling^34,36–39^. Based on results in other tumor cell lines, we expect apoptosis to play a large role in the ROS-mediated cell death; however, other cell death pathways have been implicated with CAP, and remain to be investigated in chordoma^17,20–23^. Further investigation is required to understand the mechanisms of cell killing in chordoma more specifically.

The potential clinical application of CAP in the management of chordoma is primarily as an adjuvant intra-operative tool, whereby CAP is applied to the surgical resection cavity to target residual tumor (in cases of incomplete resection) and microscopic infiltration along the surgical margins. Non-invasive transdermal application of CAP could be feasible for sacral chordomas, and could conceptually be delivered endonasally to clival chordomas. Transdermal CAP has been shown to decrease tumor volume and improve survival in various solid tumor mouse models^16,40^. CAP has also been shown to increase sensitivity to radiotherapy and could thereby serve as a radiosensitizer in tumor types where adjuvant radiotherapy is part of the current standard of care, such as in chordoma.

This study provides only in vitro evidence of cytotoxicity and ROS accumulation. Preclinical in vivo studies are required to better determine the translational potential of CAP in the care of chordoma patients. This study is also limited in that we study only the direct application of CAP. Indirect means of CAP application may also warrant study prior to any translational development.

## Conclusions

CAP is a promising therapeutic agent against chordoma, a challenging, aggressive, and deadly malignancy with limited treatment options. CAP has been shown to selectively induce cell death in various cancer types. The cell death rates observed across three different chordoma cell lines after CAP treatment in vitro suggest that chordoma may be particularly susceptible to mechanisms by which CAP mediates cytotoxicity. One of those mechanisms appears to be through the accumulation of intracellular ROS, as we have demonstrated. These in vitro studies provide strong support for further study of CAP as a treatment modality for chordoma.

## Electronic supplementary material

Below is the link to the electronic supplementary material.

Supplementary Material 1

Supplementary Material 2

## Data Availability

Data available on request from the author SP (sophie.peeters0@gmail.com).
