# Supplementary material for: Cold atmospheric plasma effectively kills chordoma cells through induction of intracellular reactive oxygen species
Source: Sci Rep. 2025 Jul 1;15:20838. doi: 10.1038/s41598-025-05916-y (PMC12219751; doi:10.1038/s41598-025-05916-y)
Supplement: Supplementary file 1 — Supplementary Material 1 [file 41598_2025_5916_MOESM1_ESM.pdf]

Cold Atmospheric Plasma Effectively Kills Chordoma Cells Through Induction of Intracellular Reactive Oxygen Species

Sophie Peeters*, MD[1], Peter B. Wu, MD[1], Blake Haist, MS[2,3], Amber Armellini, BS[1], Wi Jin Kim, MD[1], Zhitong Chen, PhD[2], Richard Obenchain, BS[2,3], George Ayad[1], Weihong Ge, PhD[4], Aparna Bhaduri, PhD[1,4], Graeme Sabiston, BS[2], Robert M. Prins, PhD[1], Richard Wirz, PhD[2,5], Anthony C. Wang*, MD[1]

[1]Department of Neurosurgery, David Geffen School of Medicine, University of California Los Angeles, Los Angeles, CA
[2]Department of Mechanical and Aerospace Engineering, University of California Los Angeles, Los Angeles, CA
[3]Department of Mechanical, Industrial, and Manufacturing Engineering, Oregon State University, Corvallis, OR
[4]Department of Biological Chemistry, David Geffen School of Medicine, University of California Los Angeles, Los Angeles, CA
[5]College of Engineering, Oregon State University, Corvallis, OR

*Corresponding authors:
Anthony C. Wang and Sophie Peeters
University of California Los Angeles, Los Angeles, CA
ACWang@mednet.ucla.edu and Sophie.Peeters0@gmail.com

Supplementary Figure

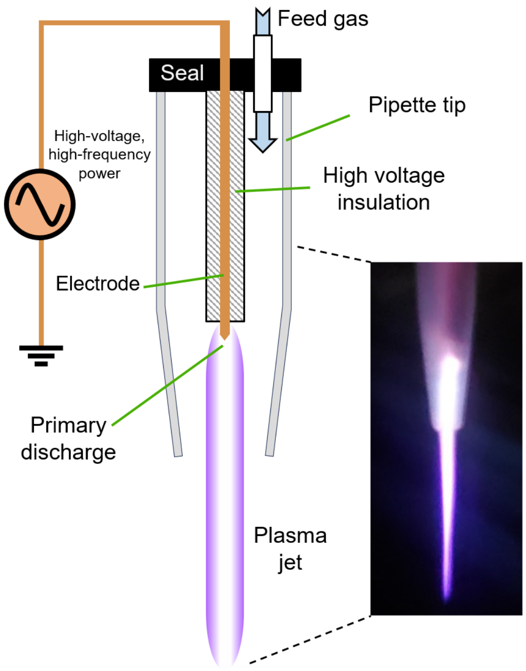

Fig 1. Single electrode CAP device functional diagram with inset during free-space operation.
