# Supplementary figures and images for: Cold atmospheric plasma effectively kills chordoma cells through induction of intracellular reactive oxygen species
Source: Sci Rep. 2025 Jul 1;15:20838. doi: 10.1038/s41598-025-05916-y (PMC12219751; doi:10.1038/s41598-025-05916-y)

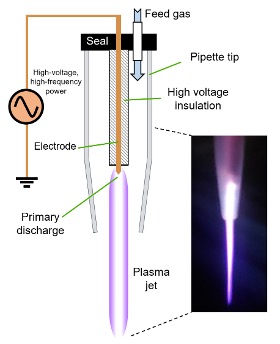

Supplement: Supplementary file 2 — Supplementary Material 2 [file 41598_2025_5916_MOESM2_ESM.jpg]
